# A Review on Edible Coatings and Films: Advances, Composition, Production Methods, and Safety Concerns

**DOI:** 10.1021/acsomega.3c03459

**Published:** 2023-08-02

**Authors:** Anam Matloob, Hudda Ayub, Muhammad Mohsin, Saadia Ambreen, Faima Atta Khan, Sadaf Oranab, Muhammad Abdul Rahim, Waseem Khalid, Gulzar Ahmad Nayik, Seema Ramniwas, Sezai Ercisli

**Affiliations:** † National Institute of Food Science & Technology, University of Agriculture, Faisalabad, 38000, Pakistan; ‡ University Institute of Food Science and Technology, The University of Lahore, Lahore 54000, Pakistan; § Department of Food Science, Faculty of Life Science, Government College University Faisalabad, Faisalabad 38000, Pakistan; ∥ Department of Biochemistry, Faculty of Life Sciences, Government College University, Faisalabad 38000, Pakistan; ⊥ Department of Food Science & Technology, Government Degree College Shopian Gagran 192303, Jammu and Kashmir, India; # University Centre for Research and Development, Chandigarh University, Gharuan, Mohali 140413, Punjab India; @ Department of Horticulture, Faculty of Agriculture, Ataturk University, Erzurum 25240, Turkey; △ HGF Agro, Ata Teknokent, TR-25240 Erzurum, Turkey

## Abstract

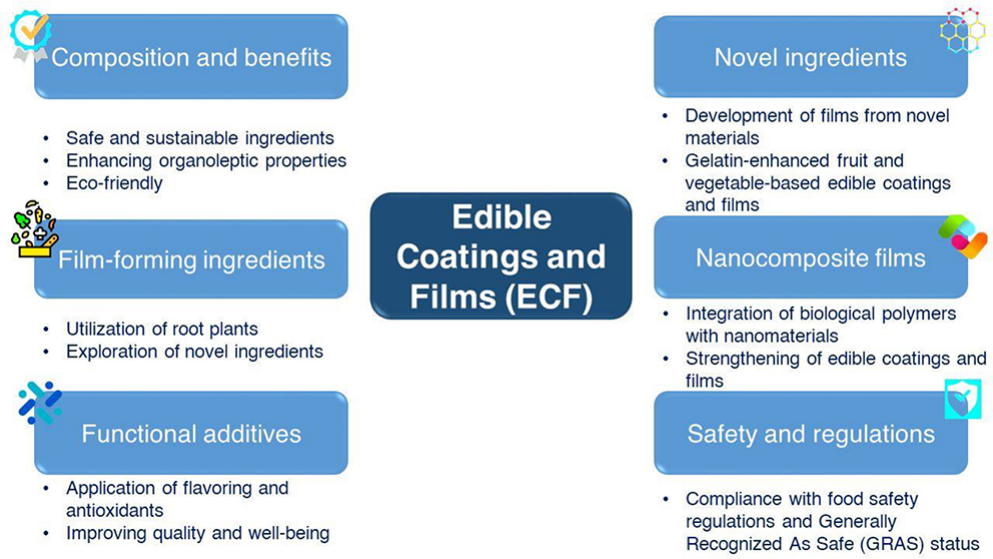

Food is a crucial source for the endurance of individuals, and quality concerns of consumers are being raised with the progression of time. Edible coatings and films (ECFs) are increasingly important in biobased packaging because they have a prime role in enhancing the organoleptic characteristics of the food products and minimizing the spread of microorganisms. These sustainable ingredients are crucial for a safer and healthier environment. These are created from proteins, polysaccharides, lipids, plasticizers, emulsifiers, and active substances. These are eco-friendly since made from innocuous material. Nanocomposite films are also beginning to be developed and support networks of biological polymers. Antioxidant, flavoring, and coloring compounds can be employed to improve the quality, wellbeing, and stability of packaged foods. Gelatin-enhanced fruit and vegetable-based ECFs compositions have the potential to produce biodegradable films. Root plants like cassava, potato, and sweet potato have been employed to create edible films and coatings. Achira flour, amylum, yam, ulluco, and water chestnut have all been considered as novel film-forming ingredients. The physical properties of biopolymers are influenced by the characteristics, biochemical confirmation, compatibility, relative humidity, temperature, water resistance, and application procedures of the components. ECFs must adhere to all regulations governing food safety and be generally recognized as safe (GRAS). This review covers the new advancements in ECFs regarding the commitment of novel components to the improvement of their properties. It is expected that ECFs can be further investigated to provide innovative components and strategies that are helpful for global financial issues and the environment.

## 1 Introduction

Biobased packaging acquired much importance in the past few years.^1^ Edible films are narrow sheets applied to products.^2^ This is made from eco-friendly safe material. Edible coatings are produced by a number of different techniques and have a great impact on preserving the nutrients such as flavoring agents, antioxidants, and antimicrobials.^2^ Functionality largely depends on their permeability, solubility, and mechanical properties. Employment of edible coatings and films (ECFs) is not a brand new technique; it has been used since prehistoric times. ECF is a comprising technique with a positive effect on quality conservancy.^3^

A partial barrier against moisture retention can be provided by an edible coating.^4^ ECFs are made from entirely sustainable and safe materials.^5^ Selection of sustainable materials for ECFs have a great impact on the formation of efficacy and user adequacy.^6^ Increasing consumer demand for food preservation from biological sources has led to modified techniques including ingestible biopolymers. Proteins, polysaccharides, and lipids might be considered the main ingredients in the synthesis of ECFs. A combination of the insignificant materials as plasticizers, emulsifiers, and vigorous ingredients, specifically antimicrobial and antioxidants, can be employed to enhance their efficacy. The processing of the commodities comes after harvesting, and packaging is a crucial step that is required to maintain the quality parameters of the products in terms of their organoleptic properties, physio-chemical properties, and sensory properties. Packaging also helps to improve distribution and transportation of the commodities. Food packaging protects the food from any kind of external damage without interacting with the internal environment of the product. External damage would be any kind of physical damage (shocks), chemical damage (temp, pH), and biological damage (damage caused by micro-organisms, insects, and animals).^7^ Most of the fruits and vegetables are highly perishable and some climacteric fruits which undergo their ripening process during and even after harvesting have shorten shelf life and are more prone to microbial attack, oxidation, and browning. To address these issues, food packaging of mainly the petroleum-based plastic packaging is extensively used but these are nondegradable and are not environmentally friendly. Biodegradable packaging materials have gained researchers interest as they are food-grade, environmentally friendly, and can be consumed with the product. Chitosan-based edible coatings have good antimicrobial, antifungal properties, and film-forming properties. In order to maintain and enhance the safety and quality characteristics of the products, chitosan, a fundamental coating material made of proteins and polysaccharides, is being employed as an alternative to plastic packaging.^8^

Dextran- and chitosan-based biopackaging had been used for mushrooms (*Agaricus bisporus*). These films exhibit good mechanical attributes in term of moisture resistance and gas permeability. These films also have antimicrobial potential, are made from safe material, and are biodegradable. Dextran and chitosan are generally recognized as safe material and are safe renewable material. These materials do not impart undesirable odor and taste to food materials. These films also exhibit good transparency and allow the visual inspection of food commodities. Dextran- and chitosan-based films help to enhance the quality and extend the shelf life of mushrooms (*Agaricus bisporus*).^9^ Characterization of oligodextran produced by *Leuconostoc mesenteroides* SF3 and its effect on film-forming properties of chitosan provide valuable insights on the potential applications of this film blend, including information about the rheological attributes, molecular weight distribution, and degree of polymerization. Oligodextran also helps to access the film-forming abilities, mechanical attributes, and functional properties of film.^10^

Edible coatings and films are used to improve the appearance of the food and promote the health of the food through environmentally friendly properties.^11^ The addition of biobased polymers also brings some challenges to some food.^12^ As fresh or lightly processed fruit and vegetables gain popularity because of their increased biological relevance and health benefits, the consumer’s demand for nonchemically preserved, superior quality foods have been steadily rising.^13^ The quality and well-being of fruits and vegetables typically diminish in the course of postharvest losses because of increased humidity content, maturing, senescence, bacteriological growth, and ecological factors.^14^ The use of edible films and coatings helps to reduce them.^13^ ECFs are employed to restore the organoleptic features of packaged foods.^15^ ECFs can also effectively reduce microbiological development in firm and semisolid food items by reducing the transmission rate of antimicrobial components from glazing material to food product. This is due to their effectiveness as discriminating barriers to gas, humidity, and solute transport.^16^

## 2 Conformation of ECFs

**Figure 1:**
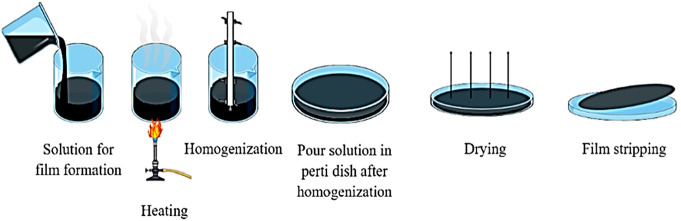
Film casting method used in the lab to produce edible films.

Currently, artificial and petroleum materials are reinstated with sustainable materials in the packing sector.^17^ Solutions with a mixture of different natural components are applied for the manufacturing of ECFs (Figure 1). ECFs are categorized based on the structural components, such as proteins, lipids, polysaccharide films, or composite materials.^18^ The mechanical properties of ECFs are influenced by the polymer matrix or compatibility of the material. The mixture of lipids and hydrocolloid mass may form a composite film. Tannic acids are mostly used in edible coating and films as a strong polyphenol, mainly extracted from different parts of plant tissues and green tea. Tannic acid has hydrogen bonding and dynamic covalent interactions between five gallol and five catechol groups, and consequently, it has higher water permeability, better mechanical strength, and powerful antioxidant, antibacterial, and antiviral properties.^19^ Tannic is used as a cross-linking agent in most edible coatings and films to improves the strength and mechanical properties because of the covalent bonds and covalent cross-linking with the coating materials. The tannic acid-based films and coating strongly reduces the weight loss, oxidation, and browning of the products.^20^ Tannic acid is generally considered safe and is biocompatible. Tannic acid has the capability to effectively cross-link polymers under mild conditions and can also cross-link with multiple functional groups.^21^ It has excellent antimicrobial and antioxidative attributes which are very helpful, but there is an issue regarding the toxicity of tannic acid.^22^ Tannic acid has pH sensitivity and is sensitive to light and oxidation. Exposure to oxygen may result in different color changes. To overcome this problem these is a need for proper handling and storage and proper packaging. pH optimization is another method to overcome this problem of tannic acid. Coating and encapsulation are potential to ways to prevent leaching and migration of tannic acid from the film.^23,24^

### 2.1 Protein-Based ECFs

**Figure 2:**
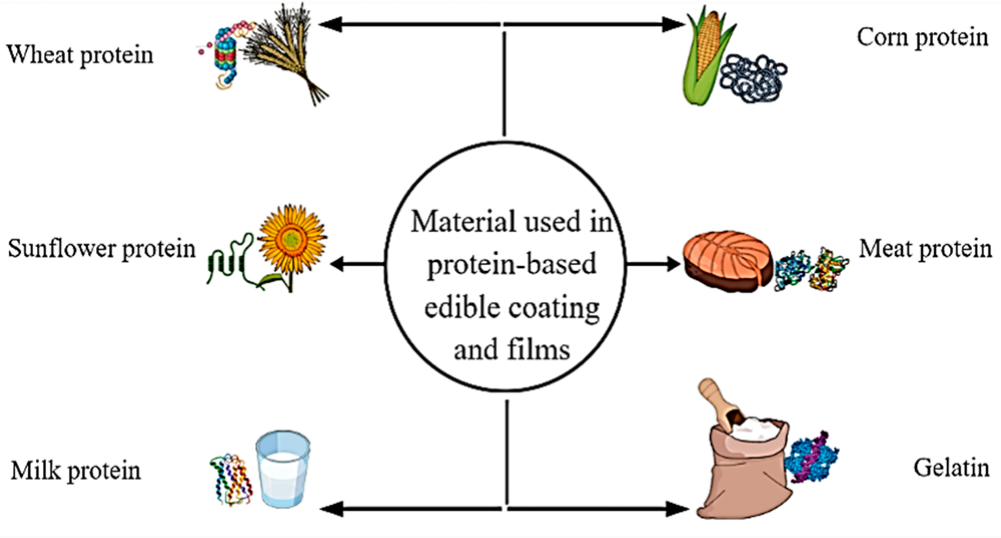
Protein-based materials used for edible coatings and films.

The specific amino acid components that make up a protein are contained in biopolymers created by deamination relapse.^25^ The protein denaturation process is done during the film formation.^5^ A resilient, interconnected, and viscoelastic film can be formed while proteins interrelate with adjacent particles by hydrogen, ionic, or covalent linkage after denaturation. The basic material for the manufacture of protein-based ECFs can be from animal or plant-based sources as shown in (Figure 2). Due to their biological value, biodegradability, and amazing film-creating abilities, biopolymers are the best option for films and coating formulations.^26^ Zein, a plant protein, can be used to benchmark infectious contaminations and decays in fruits and vegetables.^27^ Conformation, plasticity, and thermal permanence of protein are very significant to create films.^28^ Protein-based ECFs provide a tremendous barrier to gases. These biopolymer-based ECFs improved mechanical properties associated with those emerging from lipids and carbohydrates. Protein-based ECFs have significant drawbacks, including a lack of water vapor barrier components.^25^

The creation of edible films and coatings frequently uses milk proteins. Because milk includes a lot of lactose, which causes crystallization in the film, only whey protein should be used instead of entire milk proteins.^29^ Whey protein is used as an emulsifier and plasticizing agent in two forms mainly the whey proteins concentrate and whey protein isolates. Whey protein-based film is used in low humidity conditions and has high oil barrier properties and high aroma of oxygen. Whey proteins are also used in edible coatings and films as a probiotics and prebiotics source.^30^ Milk proteins are very important and valuable biopolymers. These have higher thermal stability and are nontoxic. Milk proteins have remarkable gas barrier properties because they have unique structure. Along with these beneficial characteristics there are also some downfalls of milk-based proteins which include moisture sensitivity and low elasticity. To get maximum benefits from milk-based proteins, it is advisible to select the correct milk protein source and use to appropriate reinforcing agents.^31^

Zein, a protein that can be obtained from gluten or created during the bioethanol manufacturing process, is widely employed in edible coatings and films. Zein is used with other polysaccharides and protein-based films such as with galactomannans to enhance barrier properties and imparts gloss glass surface. To improve the barrier properties, zein is used with protein-based films through the protein–protein bounding. Zein contains a balance assembly of hydrophobic and hydrolytic structures.^29^ Zein scaffolds have remarkable potential for tissue engineering. Zein protein has the ability for stability of molecules. This protein also has potential for controlled drug delivery. The zein protein have potential for usage in applications at the nano level.^32^

### 2.2 Lipid-Based ECFs

**Figure 3:**
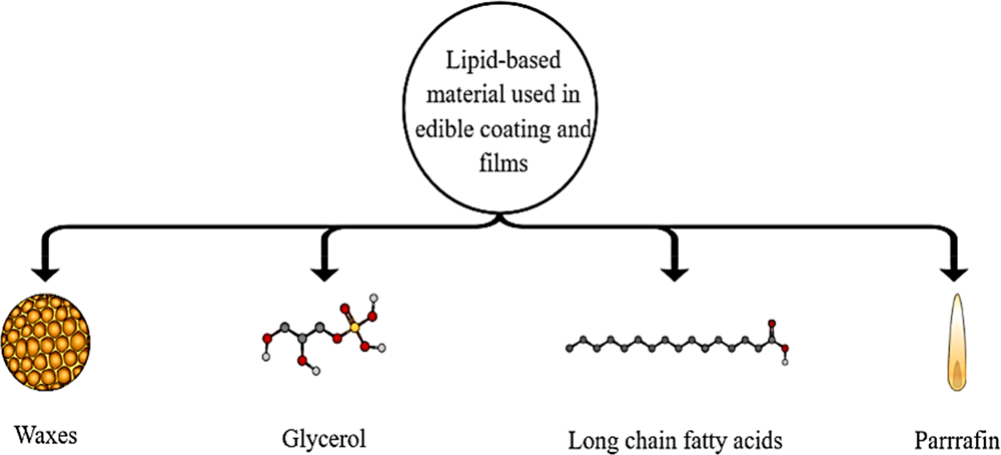
Lipid-based materials used for the production of edible coatings and films.

Hydrophobic lipids are small biological molecules, unsolvable in water. Due to hydrophobicity, lipid-based ECFs are superfluously brittle and denser. These have exceptional barriers in contradiction to moisture migration.^5^ ECFs comprising lipids in alginate extracts were manufactured by Fabra and coauthors.^33^ Materials applied in the manufacture of films and coatings comprise waxes, paraffin, and acetoglyceride, as mentioned in Figure 3. The main drawback of lipid-based ECFs is the restriction of the finished food product appearance and perception.

### 2.3 Polysaccharide-Based ECFs

**Figure 4:**
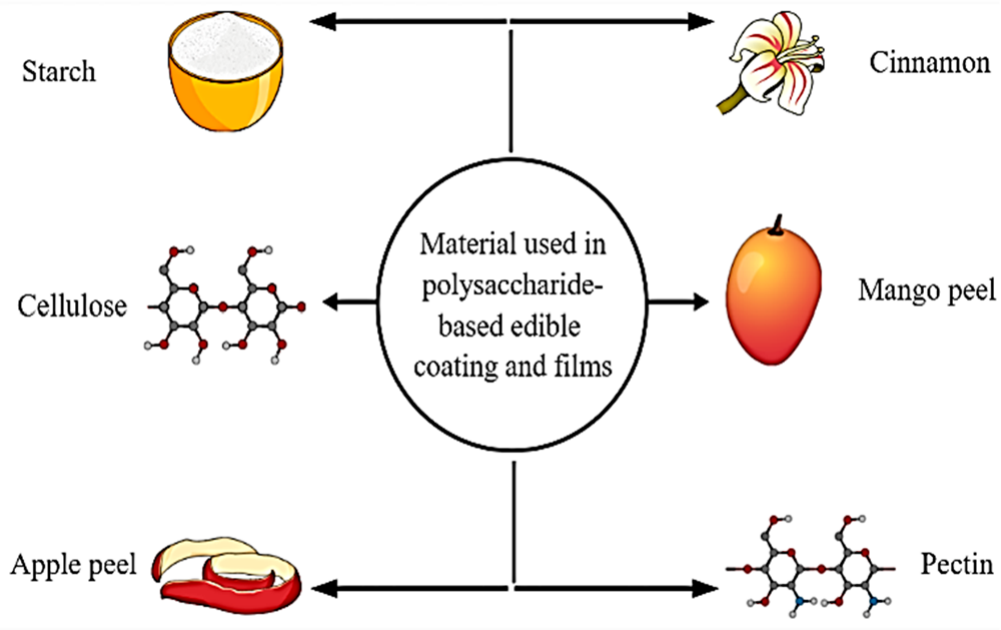
Polysaccharide-based materials used to produce edible coatings and films.

Polysaccharide-based ECFs are tough, highly soluble, colorless, and flexible because polysaccharides have linear structures.^25^ These films could be smeared in numerous food to prolong the shelf life of foodstuffs, such as fruit and vegetables, and meat (Figure 4). Polysaccharide that are mostly used in film formation are chitosan, alginate, and gums. Polysaccharide-based ECFs have noble gas impermeable properties, and effective oxygen blockers, just because of their well-organized hydrogen-bonded linkage. Incompetence as moisture barriers is their major drawback.^25^ Deacetylation of chitin by alkali solution provides chitosan. Chitosan is also obtained from invertebrates, insects, marine organisms, algae, and fungi.^34^ Chitosan has exceptional antimicrobial, antioxidant, O_2_, and CO_2_ barrier attributes.^35^ Solutions with a mixture of different polysaccharide components are applied as edible coatings on fruits and vegetables. Chitosan came up smeared in the cast of extricating or nanoparticles in biologically active layers along with peel extricate of pomegranate for the protection of fruits from fungi in 2019.^36^ Other research was conducted in which edible coatings were produced using chitosan or olive oil extracts, which prevent microbiological activity in apples and strawberries.^37^

ECFs made of algae tend to enhance the qualities and lengthen the shelf life of dairy products, cheese, meat, and other fresh produce. A study by Rangel-Marrón and co-workers assessed the impact of applying ECFs with sodium alginate to papaya blends.^38^ Another study focused on the representation of sodium alginate coatings that were reinforced using lemongrass natural ointment to be applied on fresh apple slices in order to maintain the quality of the products.^39^ Gums are hydrophilic which are tasteless, unscented, monochrome, nontoxic, eco-friendly, sustainable, and biologically safe. These characteristics of gums caused a rise in the demand for this substance.^40^ These can be arranged on the basis of their origin, shape, charge, and synthetic design.^41^

### 2.4 Composite Films

There are several drawbacks to all biopolymers used in food packaging; to address this, composite or multicomponent films are created. It comprises a blend of various materials with useful properties.^5^ A few combinations are feasible to upgrade a few obstructions and mechanical properties like protein–protein ECFs, polysaccharide-protein ECFs, and polysaccharide-lipid ECFs. At the point when ECFs with a blend of alginate besides chitosan were developed, palatability and sensory characteristics of new figs were obtained, in addition to their bioassay properties and barrier properties. Composite ECFs were also recently cast-off in another investigation to ensure the quality of grapes during storage.^42^ Nowadays, nanocomposite films are arising as one more bundling arrangement and comprise the support of the biological polymer network through nanocrystals that allow for work on their strength, isothermal, and boundary properties. This procedure is static in an undeveloped stage and is important to plan and foster improved handling innovations.^26^

### 2.5 Plasticizers

Broad connections among the biological polymers can prompt an inflexible design and fragility in the ECFs creation. It tends to be tackled by integrating plasticizing agents into preparations.^26^ Plasticizers are tiny, relatively stable particles. These substances cause the film to stretch more and lose flexibility. Because of the greater mobility of the polymeric compounds, which can limit the usage of this kind of mixture, the use of plasticizers in higher levels may hasten the arrival of the powerful complexes like antioxidants and antimicrobials present in the combination.^35^ The similarity between the compounds’ nature, size, and constituent composition is anticipated to prevent the plasticizing chemical from shifting from the film framework when they are used. The most widely used plasticizer is glycerol.^18^ Glycerol is used to lessen the brittleness of the biocomposite ECFs made from the psyllium coating.^43^ Using chitosan films, nuts, seeds, and fruits can be shielded from fungal contaminations.^44^

### 2.6 Additives

The strength, palatability, and healthful qualities of ECFs can be improved by chemical additives such plasticizers and surfactants by enhancing the utility of the package. To advance stabilizing and mechanical properties, emulsifiers can also be applied. Table 1 represents the benefits along with the properties of edible coatings. Antimicrobial, antioxidant, flavoring, and coloring agents can be engaged to boost the excellence, well-being, and stability of packaged food.^5^

**Table 1:** Beneficial Properties of ECFs

benefits	properties	refs
moisture barrier	prevent moisture loss	(45, 46)
	prevent aroma loss	
	good storage stability	
	enhance texture	
	prolog shelf life	
oxygen scavengers	control humidity	(47)
	minimize oxygen diffusion rate	
	decrease respiration	
	delay oxidation	
ethylene scavenger	extend shelf life	(48)
	lower ethylene concentration	
antimicrobial properties	minimize spoilage	(49, 50)
	control microbial growth	
	extend shelf life	
antibrowning and antioxidant properties	prevent discoloration	(51)
	control polyphenol oxidase (PPO) activity	
	strong barrier for oxygen	

## 3 Novel Materials

### 3.1 Whole Grain Flour

Material for polysaccharides protein-based ECFs acquired from ordinary crops or roots. Researchers have developed numerous systems to manufacture films from this material.^52^ Several studies have demonstrated the ability of powders derived from whole grain components to produce biofilms, similar to amaranth^53^ and quinoa^54^ or chia.^55^ Some studies reported that psyllium husk and its flour are the finest to create films. Nouraddini developed composite film from eggplant flour or corn starch.^56^ Eggplant flour has exceptional film-forming ability but diminished mechanical properties. Legume flours have worthy film-forming material just because of their high starch and protein percentage. Additionally, edible films are successfully produced from grass peas, lentils,^57^ chickpeas,^58^ and mung beans.^59^ Cassava flour-based ECFs contain abundant fiber lentil powder. These films are biodegradable or thermally stable.^60^

### 3.2 Fruit and Vegetable-Based ECFs

Formerly, fruits and vegetable blends were used in coatings. Though, they still remain a focus of research. Other than carbohydrates, the characteristics of flour are examined in the food films made from plantains and bananas.^61^ The food business generates a significant amount of food waste each year. It contains a rich measure of supplements, bioactive mixtures, or dietary strands. Pomace and seed parts of certain organic products have higher cell reinforcement action than the mash divisions.^62^*Carissa carandas* fruit polysaccharides have preservative properties. These polysaccharides have a high content of bioactive compounds and exhibit excellent antimicrobial attributes. Modification of these polysaccharides by chemical means make them suitable to use in pharmaceutical and different food products.^63^ The processing cost of organic products or vegetables relies on applied technologies.^64^ Somewhat recently, the utilization of foods grown from the ground using parts of ECFs have been a significant topic.^38^ There are innovative ECFs made from powdered substances from different mixtures, such as those starting with orange, enthused natural ingredients, or lettuce. Without the use of plasticizers, the creators obtained uniform, adaptable films, which displayed promising qualities. Potato skin flour heightened mechanical confrontation.^64^Table 2 explains all the different coating materials used for edible coatings along with their compositions. The research led to the discovery that grapefruit luminosity is a film-shaping material, with countless practical attributes for ECFs.^65^ Gelatin-enhanced film compositions created by researchers have shown incredible inventive and practical promise for making biodegradable films. Nowadays researchers are exploring innovative extraction methods to increase the yield and purity of the extracts. These techniques include gel filtration, ion-exchange chromatography, membrane filtration, enzyme-assisted extraction, microwave, and ultrasound-assisted extraction. Plant leaf-extract polysaccharides can be used as functional ingredients. Different plant leaf-extract polysaccharides have antioxidative potential.^66^ The advantages and disadvantages of cross-linking agents used in the manufacturing of ECFs are shown in Table 3.

**Table 2:** Materials Used in the Manufacturing of ECFs

no.	material	type	composition	properties	refs
01	starch	polysaccharide-based	composed of amylose and amylopectin	improve physicochemical properties	(67)
				improve optical properties	
				colorless	
				odorless	
				low oxygen permeability	
				low cost	
				oil-free appearance	
				decrease respiration rate of fruits and vegetables	
02	chitosan	polysaccharide-based	go through the process of deacetylation	antioxidative	(68)
				antimicrobial	
				nontoxic	
				biodegradable	
				biocompatible	
				microbial resistance	
				control transpiration rate	
				retard ripening process	
03	alginate	polysaccharide-based	composed of sodium salt of alginic acid	good film forming properties	(67)
				can form gels	
04	gellan gum	polysaccharide-based	consist of tetrasaccharide repetitive units	improve function properties	(67)
				good hardness	
				high transparency	
				smooth surface	
				reduction in water vapor permeability	
05	pullulan	polysaccharide-based	polysaccharide	powerful reducing agent	(67)
				increase shelf life	
06	cellulose	polysaccharide-based	linear chain polysaccharide	low water solubility	(69)
07	carboxymethyl cellulose	polysaccharide-based	consist of glucopyronosyl units	excellent oxygen barrier	(67)
				aroma barrier	
				oil barrier properties	
				antisenescence properties	
08	pectin	polysaccharide-based	heteropolymer made up of d-galacturonic acids units	water barrier properties	(70)
				resistance to breaking	
				higher thermal resistance	
09	corn zein	protein-based	prolamin protein	good film forming attributes	(67)
				moisture resistance	
10	deep eutectic solvents (DES)	solvents	hydrogen bond donor and hydrogen bond acceptor made eutectic mixture	moisture barrier	(71)
				solvent power	
				biocompatibility	
				enhanced mechanical attributes	
				versatility	
				sustainability	
				antimicrobial potential	
11	dexibuprofen-loaded silica	dispersion	ternary solid dispersions of dexibuprofen by solvent evaporation	effective anti-inflammatory properties	(72)

**Table 3:** Crosslinking Agents Used in the Manufacturing of ECFs along with Its Advantages and Disadvantages

no.	type of coating	cross-linking agents	advantages	disadvantages	refs
01	sodium alginate	Tannic acid	antioxidant and antimicrobial properties	used in high amount reduces the mechanical strength	(73)
02	glass coatings	Alcohol	UV-proofing property	development of blocking agents	(74)
03	dispersions and emulsions	Amines	high reaction rate of nucleophily	time-consuming	(75)
05	extract-based coatings	Zein fibers	encapsulate bioactive compounds, thermal stability	poor mechanical property and stability	(76)
06	protein-based coating	glutaraldehyde	food additives, cross-linked with proteins	several health hazards are associated with additives	(77, 78)
			versatility	cross-linking sensitivity	
			film integrity and stability	residual unreacted glutaraldehyde	
			water resistance		
			biocompatibility		
			stability against enzymatic degradation		
07	starch and chiton	tannic acid	decreases water vapor permeability and mechanical strength	used in high amount reduces the mechanical strength	(23, 24)
			biocompatibility	pH sensitivity	
			versatile cross-linking capabilities	light sensitivity	
			antioxidant properties	leaching	
			antimicrobial attributes	migration	
				precipitation and gelation	

### 3.3 Root Plants

Cassava, potato, and sweet potato have been used in the manufacturing of edible films and coatings. Novel film-forming materials were assessed utilizing achira flour^79^ or amylum from canna lily (*Canna indica*),^80^ yam (*Dioscorea alata*), ulluco (*Ullucus tuberosus*),^81^ or water chestnut.^82^ These films lack mechanical characteristics.

### 3.4 Plant Gums

Gum acacia is unique and is of the most outstanding, acknowledged among every single normal gum. It has worthy blending, soothing, gelling, and binding characteristics.^83^ Gum Acacia has been used in edible coating for improving functionality and emulsion stability like in pecan nuts. Coating of tomato with gum acacia presented a critical postponement in relation to variety, bulk reduction, immovability, titratable acidity, and rot rate.^84^ The same outcomes are observed in the cases of apples^85^ and strawberries.^86^ Almond gum is obtained through the bough of an almond tree. Exudate gums have the best physical, chemical, and biological properties to use as a coating.^87^ ECFs developed from *Cajanus cajan* (*Cajanus cajan* (L) Millsp) seeds, gum, and protein detach to expand the quality of coated strawberries.^88^

### 3.5 Wild Plants

Polysaccharides utilized in the ECFs are mostly amylum and fiber subordinates. A hydrophyte that could be used to obtain an ECF is the Opuntia cactus.^89^ Opuntia cactus inferred polysaccharides were applied to citrus organic products as an eatable covering. ECFs from *Pereskia aculeata* Miller mucilage leaves were developed.^90^ These films were elastic, dark in color, and plane exterior films. Used for chocolate or coffee beans. To improve the storage duration of beef slices,^91^ an edible coating from Shirazi balangu (*Lallemantia royleana*) seed was developed. Kim created edible films involving gulfweed in the two types of coverage for the utilization of smoked salmon.^92^

### 3.6 Deep Eutectic Solvents (DES)

Dep eutectic solvents (DES) are new generation green additives. These helps to improve the different properties of films, especially chitosan-based films.^93^ These solvents have excellent beneficial attributes including plasticization, improved mechanical attributes, enhanced thermal stability, and solubility enhancement. DES also have antimicrobial attributes that help to extend the shelf life of food materials.^71^ Application of DES for chitosan-based films is a recent research area. DES are safe chemical substances and have been recently used in the preparation of films because of better barrier attributes. DES help to preserve the sensory properties of food commodities.^94^ These green additives can also act as the carrier for the bioactive compounds delivery. These are also environmentally friendly substances.^95^ Nowadays, research is more focused in this area and more exploration is needed.

## 4 Physical Properties

Important attributes of biopolymers are the foundation, mechanical arrangement of the chain, and handling methodology. So in the application of biological polymers, it is vital to consider their physical and functional properties.^96^ Biopolymer films’ and coatings’ physical properties are determined by the components’ features. Biochemical confirmation and compatibility of all components are imperative to biopolymer properties. Relative humidity and temperature are also vital. The difference in water humidity stands out more clearly, and the amount of water vapors that permeate a film is more significant. Synthetic coatings have lower water vapor penetrability as compared to other films. Water resistance is impacted by film thickness.^96^

## 5 Application Methods for ECFs

The technique and way of coating application are very imperative. The suitable technique assures the thorough casing of the product and subsequently prolongs the shelf life.^97^ Different strategies for their application are brushing, individual wrapping, spreading, and fluidized-bed handling. The fundamental strategies utilized for food covering are spraying and dipping. ECFs should be consistent and free of faults.^98^

### 5.1 Dispersion and Electrostatic Dispersion

**Figure 5:**
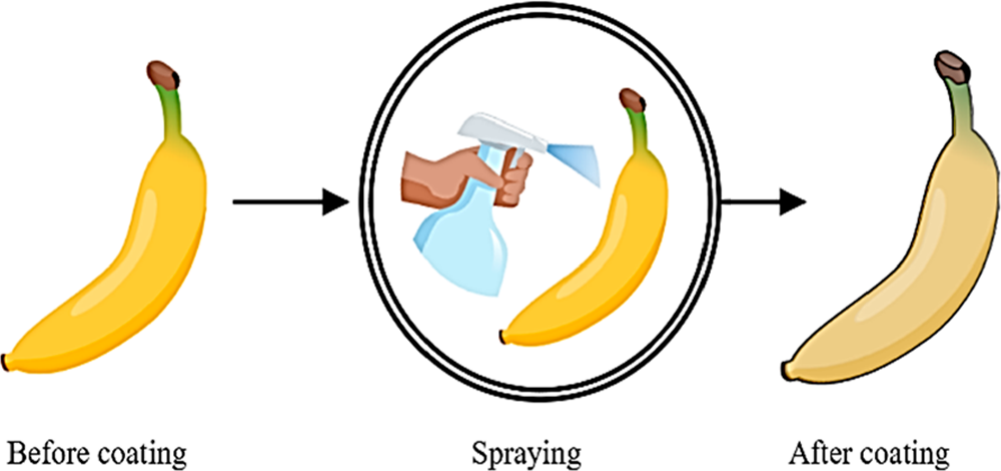
Application of coating by dispersion technique.

The technique encompasses a drizzle of the product’s whole surface with a coating solution, a less viscous solution used with a pressure of 60–80 psi.^99^ Dispersion is done by using a spray gun.^100^ The standard spray method produces fine spray with a 20 μm drop size. The electrostatic dispersion method generated constant units. Critical features for film formation comprise desiccating period, temperature, and technique. To achieve even coating spraying, cogitate is the most effective^79^ (Figure 5).

**Table 4:** Application of ECFs on Selected Food Products

no.	food product	coating materials	impact on food product	refs
1	mango	glycerol	prevent loss of firmness and weight	(105)
		carnauba wax	reduce color changes	
		aloe vera	reduce changes in pH and Brix value	
			control the rate of respiration	
2	guava	alginate	shelf life enhancement	(106)
		chitosan		
3	avocado	carboxyl methylcellulose (CMC)	prevent firmness loss and weight loss	(6)
			antimicrobial properties	
			shelf life enhancement	
4	tomatoes	pectin	lower weight loss	(6)
			shelf life enhancement	
5	strawberries	beeswax	fungal infection prevention	(107)
		chitosan	reduced weight loss	
			lowers the respiration rate	
6	figs	chitosan	improve antioxidant properties	(108)
			delaying in color change	
			*alternaria alternata* growth was repressed	
7	bell pepper	chitosan	no modification in flavonoids and antioxidant capacity	(109)
			slow growth of *Alternaria alternata*	
8	kiwi (freshly cut)	mucilage of cactus pear	improve flavor	(110)
			visual quality enhanced	
			shelf life extension	
9	blueberries	alginate	improved sensory quality	(111)
		chitosan	shelf life extension	
		apple fiber	delayed ripening	
		orange fiber	maintenance of flavor, texture, and appearance	
			chitosan	
			calcium caseinate	
			alginate	
			semperfresh	
10	jackfruit (freshly cut)	xanthan gum	microbial growth repressed	(112)
			shelf life extension	
11	pineapple (freshly cut)	alginate	quality conservation	(113)
			shelf life extension	
12	apples (freshly cut)	carboxymethyl cellulose (CMC)	shelf life extension	(114)
		chitosan	antioxidant capacity of vitamin C was maintained	
		alginic acid and gellan gum	quality enhancement	
		fiber from apple and pectin		
13	potatoes	locust bean gum	microbial growth controlled	(115)
			nutritional quality maintained	
14	watermelon (freshly cut)	alginic acid	improved texture	(116)
		polysaccharide (pectin)	shelf life extension	
			calcium lactic acid	
15	fresh-cut mangoes	alginate	browning delayed	(117)
			shelf life extension	
16	saffron	maltodextrin	improvement of physicochemical properties	(118)
		nanocellulose		
17	taro corms	chitosan	quality improvement	(119)
		starch	microbial growth inhibited	
			shelf life extension	
18	cherry tomatoes	hydroxypropyl	reduction in fungal growth	(120)
		methylcellulose	*botrytis cinerea* growth was reduced	
		beeswax		
19	mushrooms	alginic acid	shelf life extension	(121)
20	leaves of spinach	agar gewl	shelf life extension	(122)
		carrageenan		
21	asparagus (white)	na	reduction in weight loss	(123)
		carboxymethyl-cellulose (CMC)	quality preserved	
		isolate of whey protein		
22	sausages	maltodextrin	shelf life extension	(124)
		alginate	reduction in weight loss	
		carboxymethyl cellulose (CMC)		
		gelatin		
		carrageenan		
23	butter	low-density polyethylene	increased antimicrobial properties	(125)
			increased antioxidant capacities	
			shelf life extension	
24	bread	pectin	lower moisture content	(126)
		alginate	shelf life extension	
		whey protein		
		starch		
25	chicken meat	powder of peel of mango	shelf life extension	(127, 128)
		arabic gum	bioactive compounds were improved	
		PE low density	antimicrobial capacity was improved	
26	slices of ham	starch from cassava tuber	storage-life extension	(129)
		polysaccharide (chitosan)		
27	chicken nuggets	alginate	microwave heating was enhanced	(130)
28	Chicken breast (freshly cut)	carrageenan	reduction in *Campylobacter jejuni*	(131)
		polysaccharide (chitosan)	shelf life extension	
29	cheese	galactomannan	shelf life extension	(132)
		chitosan		
30	bream fish	alginate	bacterial growth inhibited	(133)
			enhanced sensory values	
			shelf life extension	
31	turkey (pouched)	alginic acid	growth of microorganisms was inhibited	(134)
		carrageenan		
		polysaccharide (starch)		

### 5.2 Immersion Technique

**Figure 6:**
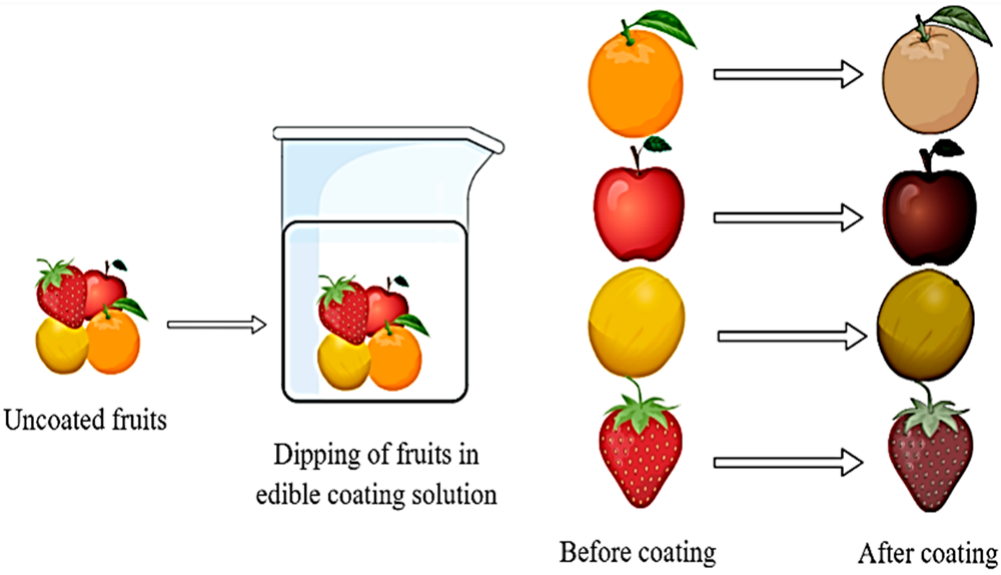
Application of coating by immersion technique.

In the immersion technique method, covering is smeared on by plunging the food item in ECF solution (Figure 6). It is an applicable technique for products with uneven surfaces. By this technique, dense coatings can be acquired. Biopolymer coating can be affected by the density and tackiness of the coating solution.^97^ In dipping, food is straightforwardly submerged into a solution and released subsequently following a certain period.^99^ The period is generally 5 to 30 s, after which the product is dried naturally. Optimal regularities can be acquired by using this technique. The drawback of this method includes hindrance in reparation due to thick coating or coating solution and can lower the functionality of food surface by weakening the outer covering. In the implementation of the ECFs, it is challenging to accomplish suitable adhesion properties.

## 6 Well-Being Issues, Protocols, And Legislation

ECFs ought to follow all expected guidelines appropriate to foodstuff constituents because these are a fundamental measure of the eatable serving.^101^ All ECFs producing components, or any effective additions, should be generally recognized as safe (GRAS) to maintain product safety and ingestion prominence. Some edible films comprise materials that can probably cause hypersensitive responses. Therefore, regardless of how little is used, an ECF containing a significant toxin should also be announced to the customer and a suitable warning should be given.^102^ Every component used in coatings should be approved by users based on their sensory perception.^103^ Additionally, the toxicity and genotoxicity of ECFs should be taken into account during assembly as yet another important factor. In such circumstances, organic oils that are frequently used in ECFs as a germicidal complex, although being defined or enrolled as GRAS by the European Commission and the United States, may introduce hypersensitive effects.^100^ There are rules for adding antioxidants and antimicrobials that are applicable to food compositions.^104^Table 4 explains the applications of EFCs on different food products.

## 7 Conclusion

In conclusion, edible films and coatings have emerged as a promising method to raise food quality, enhance shelf life, and reduce environmental waste. They frequently consist of natural polymers such as proteins, polysaccharides, and lipids and have several benefits, including the capacity to preserve food freshness, halt moisture loss, and prevent microbial contamination. They also provide an alternative to traditional packaging materials, which are frequently nonbiodegradable and contribute to plastic litter. Edible films and coatings have the potential to decrease food waste by keeping the freshness and safety of perishable foods. They also create a barrier to oxygen, moisture, and other outside elements, slowing the deterioration process and extending product shelf life. The growing consumer desire for organic and sustainable food items is another significant factor, as they are made from renewable resources and are simple to incorporate into the current food manufacturing processes. The exploration of novel materials and future advancements in formulation techniques are anticipated to define the trend toward edible films and coatings. Researchers and industry experts are expected to concentrate on improving these films’ and coatings’ mechanical qualities, barrier capabilities, and stability in order to boost their usefulness and acceptability for usage in a variety of food applications. Additionally, efforts to develop edible films and coatings with enhanced antibacterial properties are likely to continue. The production of edible films and coatings using nanotechnology appears to be a potential area for future study. Food packaging and preservation will probably become more environmentally friendly and sustainable when these materials see increasing advancements and are used widely in the food industry.
